# Immune-related gene index predicts metastasis for prostate cancer patients undergoing radical radiotherapy

**DOI:** 10.1186/s40164-022-00367-x

**Published:** 2023-01-12

**Authors:** Dechao Feng, Weizhen Zhu, Xu Shi, Zhihong Wang, Wuran Wei, Qiang Wei, Lu Yang, Ping Han

**Affiliations:** Department of Urology, Institute of Urology, West China Hospital, Sichuan University, Guoxue Xiang #37, Chengdu, 610041 Sichuan People’s Republic of China

## Abstract

In this study, we established a novel immunologic gene prognostic index (IGPI) to predict metastasis and provided new insights into tumor immune microenvironment (TIME) for PCa patients receiving radical radiotherapy. GBP2 and IGF1 were independent factors associated with metastasis-free survival. IGPI score was calculated based on GBP2 and IGF1 and this score was an independent risk factor for PCa patients undergoing radical radiotherapy. Patients with higher IGPI score were at higher risk of metastasis and biochemical recurrence, which were externally validated in the TCGA database and other GEO datasets. IGPI score had demonstrated moderate diagnostic ability of radiation resistance (AUC: 0.889). This score increased with the augment of Gleason score and T stage, as well as biochemical recurrence. Using EPIC, ESTIMATE and immunophenoscore (IPS) algorithms, cancer associated fibroblasts (CAFs), macrophages, stromal score, and estimate score were significantly higher in patients with metastasis group compared to their counterpart. Besides, for CAFs, macrophages, stromal score, and estimate score, patients with higher scores were at higher risk of metastasis, and the HRs were 3.65, 4.01, 4.27, and 3.78, respectively. IGPI score was highly positively associated with stromal score (coefficient: 0.39), immune score (coefficient: 0.43), estimate score (coefficient: 0.45), CAFs (coefficient: 0.42) and macrophages (coefficient: 0.42), while showing the opposite relationship with tumor purity (coefficient: − 0.45). In conclusion, we found that IGPI based on GBP2 and IGF1 might serve as a biomarker predicting metastasis for PCa patients. Besides, the current data further highlight the importance of CAFs in the metastatic process of PCa.

**Supplementary Information:**

The online version contains supplementary material available at 10.1186/s40164-022-00367-x.

**Dear editor,**

Among genitourinary cancers, prostate cancer (PCa) has seen the greatest increase in global incident cases over the last 30 years [1]. There has been growing recognition of the vital links between PCa and immunotherapy, including checkpoint inhibitors, cytokines, and therapeutic cancer vaccines [2]. We previously developed an immune-related gene signature to predict progression and immune infiltration for PCa patients undergoing radical prostatectomy [3]. However, radical radiotherapy, as another preferred treatment for intermediate or high-risk localized patients, is rarely studied. About 30% of patients might undergo recurrence as a result of metastasis disease, radioresistant clones and unsatisfactory treatments [4]. In this study, we remedied these problems through establishing a novel immunologic gene prognostic index (IGPI) to predict metastasis and provides new insights into tumor immune microenvironment (TIME) for PCa patients receiving radical radiotherapy. We provided a full-text article in the Additional file 1 for the detailed methods and materials used in this study.

182 differentially expressed genes (Fig. 1A), 4519 tumor-related genes through weighted gene co-expression network analysis (Fig. 1B) and 2660 immune-related genes were used to identify 21 candidate genes (Fig. 1C). Univariate and multivariate COX regression analyses indicated that GBP2 and IGF1 were independent factors associated with metastasis-free survival (Fig. 1D). IGPI score was calculated based on GBP2 and IGF1 and this score was an independent risk factor for PCa patients undergoing radical radiotherapy (Fig. 1E). IGPI score presented certain diagnostic accuracy for metastasis (Fig. 1F). We divided PCa patients according to the median value of IGPI score and patients with higher IGPI score were at higher risk of metastasis (HR: 9.57; 95%CI: 3.23–28.33; Fig. 1G) and biochemical recurrence (BCR) (HR: 3.83; 95%CI: 2.22–6.61; Fig. 1H). In the GSE21034 [5], IGPI score also presented certain value of diagnostic accuracy (AUC: 0.802; 95%CI: 0.702–0.902; Fig. 1I). In addition, patients with higher IGPI score had higher risk of metastasis (HR: 3.85; 95%CI: 2.20–6.74; Fig. 1J) in the GSE134051 [6] and progression (HR: 1.62; 95%CI: 1.08–2.44; Fig. 1K) in the TCGA database. IGPI score had demonstrated moderate diagnostic ability of radiation resistance (AUC: 0.889) in the GSE53902 [7]. This score increased with the augment of Gleason score (Fig. 1M) and T stage (Fig. 1N), as well as BCR (Fig. 1O).

**Fig. 1:**
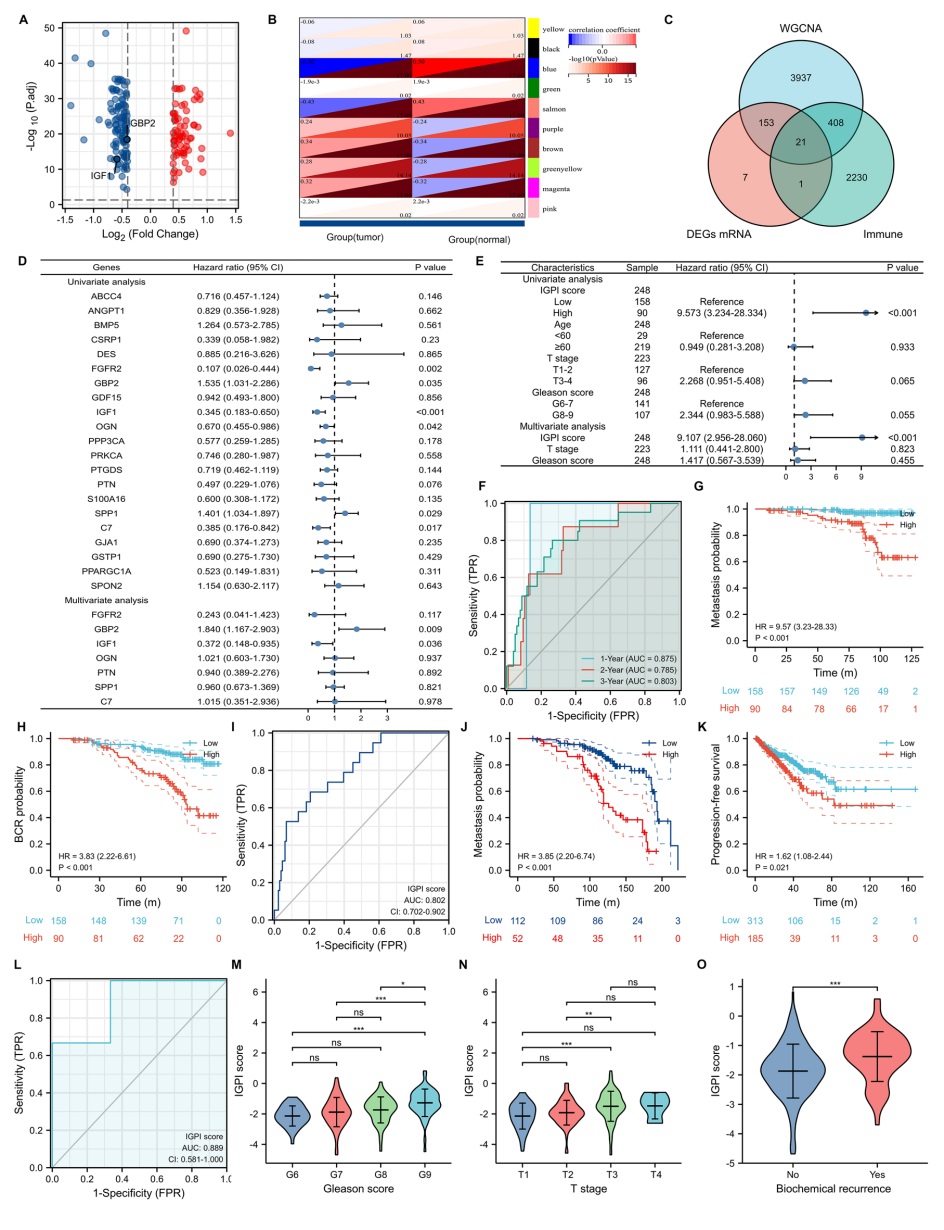
Identification of IGPI and its clinical applications. **A** volcano plot showing differentially expressed genes between tumor and normal samples; **B** modules and phenotype showing the tumor-related genes; **C** Venn diagram showing the intersection of differentially expressed genes, tumor-related genes and immune-related genes; **D** univariate and multivariate COX regression analysis of candidate genes; **E** COX regression analysis of clinical and genomic parameters; **F** time-dependent ROC curve of IGPI score; **G** analysis of metastasis free survival between high and low risk groups in the GSE116918; **H** analysis of biochemical recurrence free survival between high and low risk groups in the GSE116918; **I** ROC curve showing the diagnostic ability of metastasis in the GSE21034; **J** analysis of metastasis free survival between high and low risk groups in the GSE134051; **K** analysis of progression free survival between high and low risk groups in the TCGA database; **L** ROC curve showing the diagnostic ability of radiation resistance in the GSE53902; **M** comparison between Gleason score and IGPI score; **N** comparison between T stage and IGPI score; **O** comparison between biochemical recurrence and IGPI score. IGPI = immunologic gene prognostic index; ROC = receiver operating characteristic. ns, p ≥ 0.05; *p < 0.05; **p < 0.01; ***p < 0.001

Gene set enrichment analysis indicated that high risk group was positively associated with apoptosis, circadian rhythm, cell cycle, T cell receptor signaling pathway, chemokine signaling pathway, mismatch repair, homologous recombination, ECM receptor interaction, and so on, while it was negatively related to arachidonic acid metabolism, fatty acid metabolism, tyrosine metabolism, glutathione metabolism, and drug metabolism cytochrome P450 (Fig. 2A). Furthermore, we established the competing endogenous RNA network and identified the function axis of PART1/has-miR-6885-3p/IGF1 and GBP2 (Fig. 2B). Using EPIC, ESTIMATE and immunophenoscore (IPS) algorithms [8], cancer associated fibroblasts (CAFs), macrophages, stromal score, and estimate score were significantly higher in patients with metastasis group compared to their counterpart (Fig. 2C). Besides, for CAFs, macrophages, stromal score, and estimate score, patients with higher scores were at higher risk of metastasis, and the HRs were 3.65 (1.56–8.54), 4.01 (1.63–9.86), 4.27 (1.84–9.90), and 3.78 (1.58–9.01), respectively (Fig. 2C). However, tumor purity was significantly lower in metastasis group, and patients with higher score were less prone to metastasis compared to those with lower score (HR: 0.26; 95%CI: 0.11–0.63; Fig. 2C). IGPI score was highly positively associated with stromal score (coefficient: 0.39), immune score (coefficient: 0.43), estimate score (coefficient: 0.45), CAFs (coefficient: 0.42) and macrophages (coefficient: 0.42), while showing the opposite relationship with tumor purity (coefficient: − 0.45) (Fig. 2D).

**Fig. 2:**
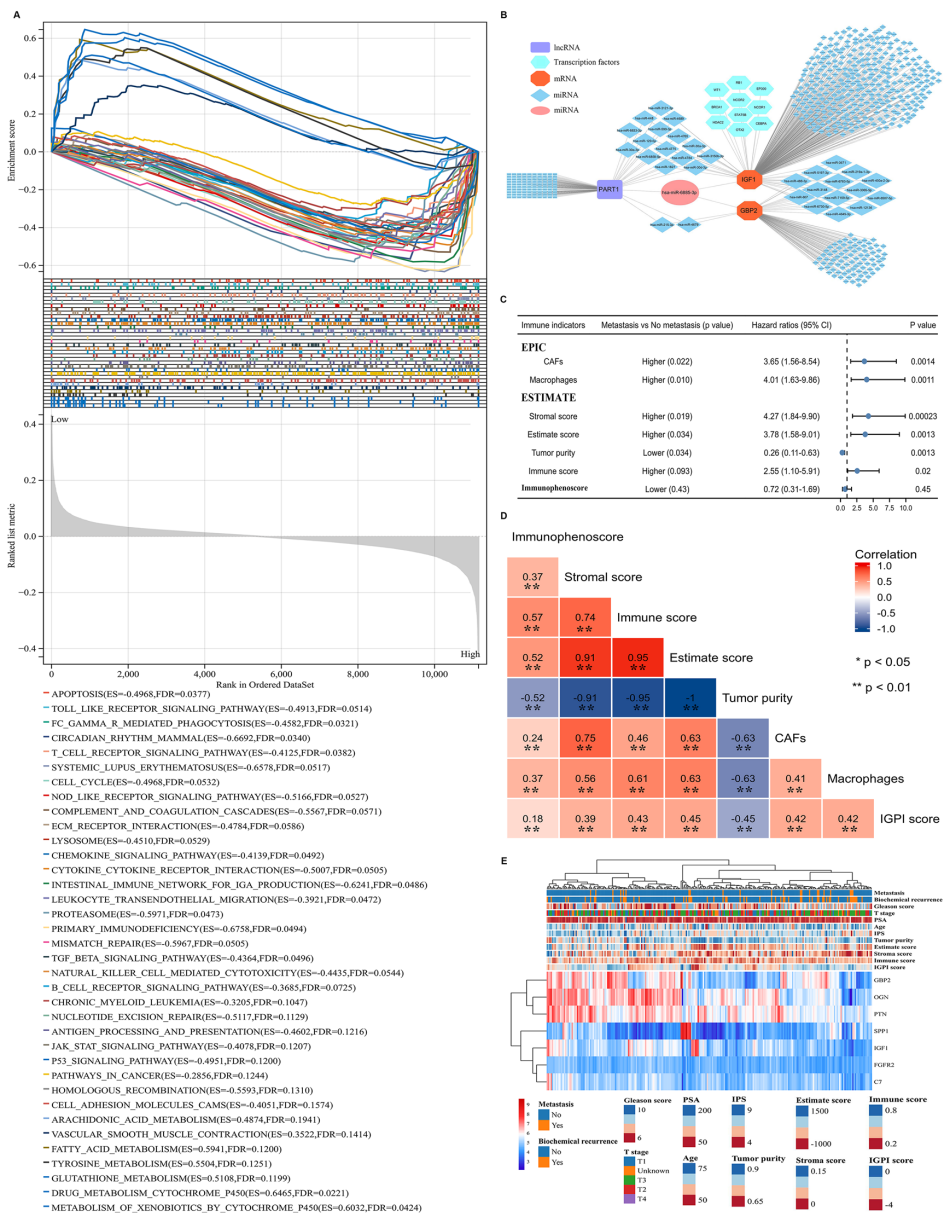
Function enrichment analysis, interaction networks and tumor immune microenvironment analysis. **A** Gene set enrichment analysis; **B** competing endogenous RNA network; **C** TIME analysis; **D** heatmap presenting the correlations among immunophenoscore, stromal score, immune score, estimate score, tumor purity, cancer associated fibroblasts, macrophages, and IGPI score; **E** heatmap of clinical information, tumor immune microenvironment parameters, IGPI score, and metastasis-related differentially expressed genes. IGPI = immunologic gene prognostic index

Experimental evidence has implicated GBP2 and IGF1 in the metastasis of other tumors, such as breast cancer [9]. We found that immune-related pathways and ECM receptor interaction might participate in the metastasis of PCa. CAFs, as the most important stromal cells, mediate ECM deposition and remodeling in the reactive stroma and increase tissue stiffness and induce mechanical stress [10]. Prostatic CAFs could induce tumorigenesis in normal human prostatic epithelial cell in vitro via the secretion of CXCL12, and this mechanism was found to be dependent on the presence of TGF-β in vivo in a mouse model, which was consistent with our findings [11, 12].

## Conclusion

We found that IGPI based on GBP2 and IGF1 might serve as a biomarker predicting metastasis for PCa patients.

## Supplementary Information

**Additional file 1.** The full-text of this correspondence.

## Data Availability

The datasets presented in this study can be found in online repositories. The names of the repository/repositories and accession number(s) can be found in the article/additional material.
