# Supplementary material for: Immune-related gene index predicts metastasis for prostate cancer patients undergoing radical radiotherapy
Source: Exp Hematol Oncol. 2023 Jan 12;12:8. doi: 10.1186/s40164-022-00367-x (PMC9835256; doi:10.1186/s40164-022-00367-x)
Supplement: Supplementary file 1 — Additional file 1. The full-text of this correspondence. [file 40164_2022_367_MOESM1_ESM.docx]

Immune-related gene index predicts metastasis for prostate cancer patients undergoing radical radiotherapy

**Running title:** An immune-related prognostic index for prostate cancer

**Authors:**

Dechao Feng^1, *^, Weizhen Zhu^1^, Xu Shi, ^1^, Zhihong Wang ^1^, Wuran Wei^1^, Qiang Wei^1^, Lu Yang^1, &^, Ping Han, ^1, &^

^1^ Department of Urology, Institute of Urology, West China Hospital, Sichuan University, Chengdu 610041, China

^*^ First author.

^&^ Corresponding Author: Lu Yang and Ping Han were co-corresponding authors, Department of Urology, Institute of Urology, West China Hospital, Sichuan University, Guoxue Xiang #37, Chengdu, 610041, Sichuan, People's Republic of China.

Tel: +86-28-85422444

Fax: +86-28-85422451

E-mail:

Lu Yang: [wycleflue@scu.edu.cn](mailto:wycleflue@scu.edu.cn);

Ping Han: [hanping@scu.edu.cn](mailto:hanping@scu.edu.cn)

**Abstract**

**Background:** To establish a novel immunologic gene prognostic index (IGPI) to predict metastasis and provide new insights into tumor immune microenvironment (TIME) for patients with prostate cancer (PCa) undergoing radical radiotherapy.

**Methods:** Four GEO datasets were used to determine IGPI through COX regression analysis. Two GEO datasets were used to external validation. Function analysis and TIME analysis were performed. All analyses were conducted with R version 3.6.3 and its suitable packages. Cytoscape 3.8.2 was used to establish network

**Results:** GBP2 and IGF1 were eventually identified to establish IGPI score. IGPI score increased with the augment of Gleason score and T stage, as well as biochemical recurrence (BCR) and prostate specific antigen (PSA). Patients with higher IGPI score were at higher risk of metastasis (HR: 9.57; 95%CI: 3.23-28.33), BCR (HR:3.83; 95%CI: 2.22-6.61), and metastasis after BCR (HR: 3.30; 95%CI: 1.12-9.77). Gene set enrichment analysis indicated that immune-related pathways, apoptosis, circadian rhythm, cell cycle, mismatch repair, homologous recombination, and ECM receptor interaction might participate in the metastasis of PCa. PDCDILG2 (namely PD-L2) mRNA expression was significantly higher in metastasis group when compared to no metastasis group. TIME analysis indicated that cancer associated fibroblasts (CAFs), tumor-associated macrophages (TAMs), stromal score, and estimate score were significantly higher in patients with metastasis group compared to their counterpart. Besides, for CAFs, macrophages, stromal score, and estimate score, patients with higher scores were at higher risk of metastasis, and the HRs were 3.65, 4.01, 4.27, and 3.78, respectively.

**Conclusion:** The evidence from this study suggests that IGPI based on GBP2 and IGF1 might serve as a biomarker predicting metastasis free survival of PCa. Besides, the current data further highlight the importance of CAFs and TAMs in the metastatic process of PCa.

**Keywords:** Immunologic gene prognostic index, prostate cancer, tumor immune microenvironment, biochemical recurrence, metastasis.

**Introduction**

Worldwide, prostate cancer (PCa) has always been a research hotspot in the field of urology as the most frequently diagnosed cancer and the most lethal urological neoplasms in aging male [1]. In addition, the increased screening strategy and changes in diet patterns results in the increasing morbidity in most Asian countries [1-3]. PCa is a typical representative of indolent tumors, for which patients are usually recommended to active surveillance avoiding the unwanted impairments from overtreatment like erectile dysfunction and lower urinary tract symptoms [4]. However, patients with intermediate or high-risk localized and locally advanced cancer are required radical prostatectomy or radiotherapy with or without androgen deprivation therapy in most instances [5]. About 30% of patients might undergo recurrence as a result of metastasis disease, radioresistant clones and unsatisfactory treatments [6-7]. It is indisputable that PCa can be classified as hormone-dependent and castration-resistant PCa (CRPC), and the latter is a crucial watershed for the prognosis of patients, accounting for approximately two-thirds of PCa-related mortalities [8-9].

In recent years, there has been growing recognition of the vital links between PCa and immunotherapy, including checkpoint inhibitors, cytokines, and therapeutic cancer vaccines [10]. Many gene signatures have been developed to predict immune infiltration and outcomes in PCa patients, but most studies focused on biochemical recurrence (BCR) of prostate specific antigen (PSA) after localized treatments [11-15]. Although BCR is a sensitive predictor of disease progression, it is not specific [16]. Moreover, rising PSA does not mean an increased risk of death from PCa for many older men, indicating that it is not a validated surrogate for PCa-related mortality [17-18]. Given the life-threatening condition of metastatic CRPC and limited clinical value of BCR, there is an urgent need to develop gene biomarkers to predict metastasis probability for PCa patients. This paper seeks to remedy these problems through establishing a novel immunologic gene prognostic index (IGPI) to predict metastasis and provides new insights into tumor immune microenvironment (TIME) for PCa patients undergoing radical radiation therapy.

**Methods**

**Data preparation**

Four GEO datasets (GSE116918, GSE32571, GSE62872, and GSE79021) with 973 samples have been developed to IGPI associated with metastasis [7, 19-21]. We used R package “inSilicoMerging” to merge the four datasets [22], and “removeBatchEffect” function of R pachkage “limma” was further used to remove the batch effect, which could be seen in our previous study [23]. Subsequently, mRNA expression matrix was extracted. GSE116918 with 249 tumor samples and complete survival information was used to identify definitive genes associated with metastasis [7]. Patients were treated with 70–74 Gy external beam radiation therapy (EBRT) in 2 Gy fractions with 3D conformal or intensity modulated techniques over 7–7.5 weeks. Node-negative patients received elective pelvic nodal irradiation at the physician’s discretion; node-positive patients had radiotherapy to pelvic nodal regions. Short (≤6 months) or long (>6–36 months) course androgen deprivation therapy commenced at least 3 months before radiation with luteinizing hormone releasing hormone agonists or antiandrogens. Other three datasets [19-21] with tumor and normal samples were used to determine candidate genes through intersection of tumor related genes via weighted gene co-expression network (WCGNA) analysis, differentially expressed genes (DEGs) and immune related genes from ImmPort and InnateDB databases [24-25]. DEGs genes were screened by llogFCl≥0.5 and padj <0.05. GSE21034 [26] and GSE134051 [27] were used to validate the diagnostic and prognostic values of IGPI score based on definitive genes. Figure 1 presented the flowchart of this study.

**Gene interaction and functional enrichment analysis**

Genomic mutations and interaction networks among transcription factor (TF), miRNA, lncRNA and mRNA were elucidated through databases of GSCALite [28], GeneMANIA [29], LncBase [30], TRRUST [31] and miRDB [32-33]. Gene ontology (GO) and Kyoto Encyclopedia of Genes and Genome (KEGG) analysis of candidate genes were conducted to explore possible biological functions and signaling pathways using the package R “clusterProfiler”. GO analysis included biological process (BP), cell composition (CC) and molecular function (MF) (P<0.05 was statistically significant). Patients of GSE116918 [7] were divided into high and low groups according to cut-off value of receiver operating characteristic (ROC) curve. For gene set enrichment analysis (GSEA), GSEA software (version 3.0) was obtained from GSEA website [34], while “h.all.v7.4.symbols.gmt”, “c2.cp.kegg.v7.4.symbols.gmt” and “c8.all.v7.4.symbols.gmt” were downloaded from molecular signature database [35] to evaluate related pathways and molecular mechanisms. Based on gene expression profile and risk groups, the minimum gene set was set as 5, and the maximum gene set was set as 5000. P < 0.05 and false discovery rate < 0.25 were considered statistically significant.

**TIME analysis**

Expression of immune checkpoint genes from GSE116918 [7] was extracted, and the expression distribution of these genes between metastasis and no metastasis was conducted. The m6A-related genes derived from Juan Xu's research [36] on the molecular characterization and clinical significance of m6A modulators across 33 cancer types. The box plot was implemented by the R software package “ggplot2”; the heat map is displayed by the R software package “pheatmap”. IOBR [37] is an immunologic tumor biology computing tool. Here, we chose EPIC [38], ESTIMATE [39] and immunophenoscore (IPS) [40] algorithms using R package “IOBR” [37] to calculate scores of immune infiltrating cells in each sample of GSE116918 [7]. Immune parameters related to differential expression and metastasis were furthered used to evaluate correlations with IGPI score. In addition, relationship among these parameters was also calculated.

**Statistical analysis**

All analyses were conducted with R version 3.6.3 (https://www.r-project.org/) and

its suitable packages. Cytoscape 3.8.2 was used to establish network [41]. R package “WGCNA” was used to perform WGCNA analysis. Metastasis free survival (MFS) was regarded as the primary outcome. Normality test was performed through Shapiro-Wilk method. The significance of two groups of samples was tested by Wilcox test, and the significance of three groups or more samples was tested by Kruskal-Wallis test. We used Spearman’s correlation analysis to describe the correlation between quantitative variables without a normal distribution. The survival analysis was conducted through Kaplan-Meier curve. Variables associated with metastasis in the univariable Cox regression analysis were included in the multivariable Cox regression models. ROC curves were conducted by R package “timeROC” and “pROC”. All the statistical tests mentioned above are two-sided. P-values of < 0.05 were considered statistically significant. Distinctive mark: ns, p≥0.05; *, p< 0.05; **, p<0.01; ***, p<0.001.

**Results**

**Baseline data and clinical values**

mRNA expression matrix of 11149 genes were obtained after combination, removing batch effects, and extraction. 4519 tumor related genes were identified from blue, salmon, brown, and magenta modules (Fig. 2B). 21 candidate genes were obtained through intersection of WGCNA analysis, DEGs and immune-related genes (Fig. 2D). Univariate and multivariate COX regression analyses indicated that GBP2 and IGF1 were independent factors associated with MFS (Fig. 2E). Therefore, we calculated the IGPI risk score through formula as follows: IGPI score=0.60721*GBP2-1.00159*IGF1. Furthermore, univariate and multivariate COX regression analyses of IGPI score and clinical indicators determined that IGPI score was an independent factor related to MFS (Fig. 2F). Figure 3G showed the protein-protein network associated with GBP2 and IGF1. We identified lncRNA PART1 (NCBI reference sequence: NR_028508.1) through screening of differential expression and association with metastasis from GSE116918 [7]. Further, we established the competing endogenous RNA (ceRNA) network and identified the function axis of PART1/has-miR-6885-3p/IGF1 and GBP2 (Fig.2H).

We used the Sankey chart to demonstrate the fluid relationship among clinical data, IGPI score and metastasis outcome (Fig. 3A). Additionally, the association among clinical information, TIME parameters, IGPI score, and metastasis-related DEGs was presented in Figure 4B. IGPI score presented certain diagnostic accuracy for metastasis (Fig. 3C-D), as well as metastasis after BCR (Fig. 3E). IGPI score increased with the augment of Gleason score and T stage, as well as BCR and PSA (Fig. 3F-I). Patients with higher IGPI score were at higher risk of metastasis (HR: 9.57; 95%CI: 3.23-28.33; Fig.3J), BCR (HR:3.83; 95%CI: 2.22-6.61; Fig.3K), and metastasis after BCR (HR: 3.30; 95%CI: 1.12-9.77; Fig.3L). For patients from GSE134051 [27], IGPI score also showed certain diagnostic accuracy (AUC: 0.603; Fig. 3M) and prognostic value (HR: 3.85; 95%CI: 2.20-6.74; Fig. 3N). Analogously, GSE21034 [26] also presented certain value of diagnostic accuracy (AUC: 0.802; 95%CI: 0.702-0.902; Fig. 3O).

**Function analysis**

GO analysis indicated that the candidate genes involved in tissue remodeling, ossification, epithelial cell proliferation, regulation of tumor necrosis factor production, collagen-containing extracellular matrix (ECM), growth factor activity, integrin binding, and cytokine activity (Fig. 4A). KEGG analysis showed that the candidate genes participated in MAPK signaling pathway, EGFR tyrosine kinase inhibitor resistance, PI3K/AKT signaling pathway, PCa, Ras signaling pathway and HIF-1 signaling pathway (Fig. 4B). GSEA analysis indicated that high risk group was positively associated with apoptosis, circadian rhythm, cell cycle, T cell receptor signaling pathway, chemokine signaling pathway, mismatch repair, homologous recombination, ECM receptor interaction, and so on, while it was negatively related to arachidonic acid metabolism, fatty acid metabolism, tyrosine metabolism, glutathione metabolism, and drug metabolism cytochrome P450 (Fig. 4C). For hallmarks, high risk group was positively associated with glycolysis, coagulation, apoptosis, PI3K/AKT/MTOR signaling, DNA repair, unfolded protein response, TGF beta signaling, epithelial mesenchymal transition, IL6/JAK/STAT3 signaling, interferon gama response, E2F targets, G2M checkpoint, interferon alpha response, and so on (Fig. 4D). For cell type signature gene sets, high risk group was positively associated with MURARO-PANCREAS-ENDOTHELIAL CELL, MURARO-PANCREAS-DUCTAL-CELL, HAY-BONE-MARROW-NK CELLS, and HAY-BONE-MARROW-MONOCYTES, while being negatively related to HAY-BONE-MARROW-CD34-POS-EO-B-MAST (Fig. 4E).

**Genomic alterations and TIME analysis**

No relationship was found between single nucleotide variation and GBP2 and IGF1. For copy number variation (CNV), GBP2 mainly involved in heterozygous deletion (8.54%), and IGF1 principally participated in heterozygous amplification (5.49%) and deletion (3.86%) (Fig. 5A). Tumor samples presented higher methylation of GBP2 and IGF1 than norm samples in prostate adenocarcinoma (PRAD) (Fig. 5E). Negative correlations were observed between methylation and mRNA expression of GBP2 and IGF1 (Fig. 5F). Patients with higher methylation of IGF1 were prone to disease and progression in PRAD compared to those with lower methylation of IGF1 (Fig. 5G).

No obvious difference of m6A-related mRNA expression was detected between metastasis and no metastasis groups (Fig. 5H). PDCDILG2 (namely PD-L2) mRNA expression was significantly higher in metastasis group when compared to no metastasis group (Fig. 5I). Cancer associated fibroblasts (CAFs), macrophages, stromal score, and estimate score were significantly higher in patients with metastasis group compared to their counterpart (Fig. 5J). Besides, for CAFs, macrophages, stromal score, and estimate score, patients with higher scores were at higher risk of metastasis, and the HRs were 3.65 (1.56-8.54), 4.01 (1.63-9.86), 4.27 (1.84-9.90), and 3.78 (1.58-9.01), respectively (Fig. 5J). However, tumor purity was significantly lower in metastasis group, and patients with higher score were less prone to metastasis compared to those with lower score (HR:0.26; 95%CI: 0.11-0.63; Fig. 5J). Figure 5K presented the correlation heatmap among IPS, stromal score, immune score, estimate score, tumor purity, CAFs, macrophages and IGPI score. IGPI score was highly positively associated with stromal score (coefficient: 0.39), immune score (coefficient: 0.43), estimate score (coefficient: 0.45), CAFs (coefficient: 0.42) and macrophages (coefficient: 0.42), while showing the opposite relationship with tumor purity (coefficient: -0.45) (Fig. 5K).

**Discussion**

It is now well established that metastatic CRPC contributes to the predominant cause of PCa-related mortality despite of the fact that most patients are in indolent condition without any threat to death [5]. The role of BCR remains largely unexamined as a surrogate of cancer specific survival and overall survival [16]. Thus, it is essential to determine factors associated with metastasis or metastasis after BCR following primary curative therapy for the sake of avoiding overtreatment for patients who might never have progression except for PSA recurrence [17, 42]. What is noteworthy is that PSA doubling time < 12 months or ≤ 7.5 months, and PSA ≥ 0.5 ng/mL are independent predictors of MFS [43]. The EAU guideline reported that ^11^C-choline positron emission tomography/computed tomography is of limited importance if PSA is <1.0ng/ml, and bone scans and computed tomography can be omitted unless PSA is >10ng/ml [44]. Given the importance of identifying metastasis state and the limitation of current clinical and radiologic indicators, this study makes an attempt to obtain data which might facilitate the process of addressing these research gaps.

GBP2 and IGF have been experimentally demonstrated involving in the metastasis of other tumors, like breast cancer, pancreatic cancer, and colorectal cancer [45-48]. In this paper, IGPI score showed comparable diagnostic and prognostic values when compared the previous PCa genomic classifies consisting of 22 markers [49]. Thus, this indicator is more convenient to daily clinical practice. Besides, we further found that PART1, a member of lncRNA, was differentially expressed and associated with metastasis of PCa, which was consistent with the previous findings [50-51]. Actually, PART1 has also been demonstrated definite links with other tumors, such as colorectal cancer [52], hepatocellular carcinoma [53], and lung cancer [54]. In the ceRNA network, lncRNA PART1 might influence the expression of GBP2 and IGF1 via modulating has-miR-6885-3p.

GSEA analysis indicated that immune-related pathways, apoptosis, circadian rhythm, cell cycle, mismatch repair, homologous recombination, and ECM receptor interaction might participate in the metastasis of PCa. For the past two decades, one of the rather surprising findings in the field of caners has been the crosstalk between tumors and their surroundings, namely TIME. TIME consists of surrounding blood vessels, immune cells, fibroblasts, bone marrow-derived inflammatory cells, various signaling molecules and ECM [55]. In this study, we observed that patients in metastasis group had higher infiltration level of CAFs and macrophages, as well as stromal score and estimate score, when compared to those in no metastasis group. Conversely, the tumor purity was lower in patients with metastasis group than their counterpart. No significant difference was detected between the two groups with regard to immune score and IPS. These findings may help us to prioritize those stromal components playing a major role in the metastasis of PCa. In the tumor microenvironment, a series of cancerogenic alterations respond to tumor cells promote the formation of reactive stroma, characterized by the presence of ‘myofibroblasts -like’ CAFs, altered ECM deposition, neovascularization and immune cell infiltration [56-57]. CAFs, as the most important stromal cells, mediate ECM deposition and remodeling in the reactive stroma and increase tissue stiffness and induce mechanical stress [56-58]. These changes and secretion properties of CAFs promotes tumor growth and progression [57]. Prostatic CAFs could induce tumorigenesis in normal human prostatic epithelial cell in vitro via the secretion of CXCL12, and this mechanism was found to be dependent on the presence of TGF-β in vivo in a mouse model, which was consistent with our findings [59]. Tumor-associated macrophages (TAMs) are major innate immune cells that constitute up to 50% of the cell mass of human tumors [60]. Majority of TAMs are programmed by tumor microenvironment to support primary tumor growth and metastatic spread [60]. Besides, Tumor-associated macrophages and tumor cells can secrete special cytokines to form a positive feedback loop to promote the formation and maintenance of tumor malignant phenotype [60]. Similar to the previous study [61], we observed that PD-L2 expression was higher in metastasis group compared to no metastasis group, indicating that PL-L2 might serve as a potential therapeutic target.

Despite these promising results, questions remain. There can be no denying that gene expression signatures are subject to sampling bias caused by intratumor genetic heterogeneity. Besides, the microenvironment features might be distinct in different tumor regions, such as tumor core and invasive margin. More importantly, all findings in this study still warranted to further external validation through large sample research, thereby exploring the deeper mechanism of pathogenesis.

**Conclusion**

The evidence from this study suggests that IGPI based on GBP2 and IGF1 might serve as a biomarker predicting MFS of PCa. Besides, the current data further highlight the importance of CAFs and TAMs in the metastatic process of PCa.

**Declarations**

**Ethical Approval and Consent to participate**

The authors are accountable for all aspects of the work in ensuring that questions related to the accuracy or integrity of any part of the work are appropriately investigated and resolved.

**Consent for publication**

Not applicable.

**Availability of supporting data**

The datasets presented in this study can be found in online repositories. The names of the repository/repositories and accession number(s) can be found in the article/supplementary material.

**Competing interests**

The authors have no conflicts of interest to declare.

**Funding**

This program was supported by the National Natural Science Foundation of China (Grant Nos. 81974099, 82170785, 81974098, 82170784), programs from Science and Technology Department of Sichuan Province (Grant Nos. 2021YFH0172), Young Investigator Award of Sichuan University 2017 (Grant No. 2017SCU04A17), Technology Innovation Research and Development Project of Chengdu Science and Technology Bureau (2019-YF05-00296-SN), Sichuan University--Panzhihua science and technology cooperation special fund (2020CDPZH-4). The funders had no role in study design, data collection or analysis, preparation of the manuscript, or the decision to publish.

**Authors' contributions**

DCF proposed the project, conducted data analysis, interpreted the data, and wrote the manuscript; WZZ, ZHW, and XS conducted data analysis, interpreted the data; PH and LY supervised the project, and interpreted the data; All authors reviewed and edited the manuscript.

**Acknowledgements**

The results showed here are in whole or part based upon data generated by the TCGA Research Network: <https://www.cancer.gov/tcga>.

**References**

1. Sung H, Ferlay J, Siegel RL, et al. Global Cancer Statistics 2020: GLOBOCAN Estimates of Incidence and Mortality Worldwide for 36 Cancers in 185 Countries. CA Cancer J Clin. 2021;71(3):209-249.

2. Feng D, Liu S, Li D, et al. Analysis of conventional versus advanced pelvic floor

muscle training in the management of urinary incontinence after radical prostatectomy:

a systematic review and meta-analysis of randomized controlled trials. Transl Androl

Urol. 2020;9(5):2031-2045.

3. Kimura T, Sato S, Takahashi H, Egawa S. Global Trends of Latent Prostate Cancer

in Autopsy Studies. Cancers (Basel). 2021;13(2):359.

4. Lowenstein LM, Basourakos SP, Williams MD, et al. Active surveillance for prostate and thyroid cancers: evolution in clinical paradigms and lessons learned. Nat Rev Clin Oncol. 2019;16(3):168-184.

5. Teo MY, Rathkopf DE, Kantoff P. Treatment of Advanced Prostate Cancer. Annu Rev Med. 2019; 70:479-499.

6. Bansal D, Reimers MA, Knoche EM, et al. Immunotherapy and Immunotherapy

Combinations in Metastatic Castration-Resistant Prostate Cancer. Cancers (Basel).

2021;13(2):334.

7. Jain S, Lyons CA, Walker SM, et al. Validation of a Metastatic Assay using biopsies to improve risk stratification in patients with prostate cancer treated with radical radiation therapy. Ann Oncol. 2018;29(1):215-222.

8. Liu CM, Kao CL, Tseng YT, et al. Ginger Phytochemicals Inhibit Cell Growth and Modulate Drug Resistance Factors in Docetaxel Resistant Prostate Cancer Cell. Molecules. 2017;22(9):1477.

9. Zhang L, Li Y, Wang X, et al. Five-gene signature associating with Gleason score serve as novel biomarkers for identifying early recurring events and contributing to early diagnosis for Prostate Adenocarcinoma. J Cancer. 2021;12(12):3626-3647.

10. Wurz GT, Kao CJ, DeGregorio MW. Novel cancer antigens for personalized immunotherapies: latest evidence and clinical potential. Ther Adv Med Oncol. 2016;8(1):4-31.

11. Luan J, Zhang Q, Song L, et al. Identification and validation of a six immune-related gene signature for prediction of biochemical recurrence in localized prostate cancer following radical prostatectomy. Transl Androl Urol. 2021;10(3):1018-1029.

12. Zhang L, Li Y, Wang X, et al. Five-gene signature associating with Gleason score serve as novel biomarkers for identifying early recurring events and contributing to early diagnosis for Prostate Adenocarcinoma. J Cancer. 2021;12(12):3626-3647.

13. Shao N, Tang H, Mi Y, et al. A novel gene signature to predict immune infiltration and outcome in patients with prostate cancer. Oncoimmunology. 2020;9(1):1762473.

14. Long X, Hou H, Wang X, et al. Immune signature driven by ADT-induced immune microenvironment remodeling in prostate cancer is correlated with recurrence-free survival and immune infiltration. Cell Death Dis. 2020;11(9):779.

15. Luan JC, Zhang QJ, Zhao K, et al. A Novel Set of Immune-associated Gene Signature predicts Biochemical Recurrence in Localized Prostate Cancer Patients after Radical Prostatectomy. J Cancer. 2021;12(12):3715-3725.

16. Pound CR, Partin AW, Eisenberger MA, et al. Natural history of progression after PSA elevation following radical prostatectomy. JAMA. 1999; 281:1591–1597.

17. Van den Broeck T, van den Bergh RCN, Arfi N, et al. Prognostic Value of Biochemical Recurrence Following Treatment with Curative Intent for Prostate Cancer: A Systematic Review. Eur Urol. 2019;75(6):967-987.

18. Mehra R, Udager AM, Ahearn TU, et al. Overexpression of the Long Non-coding RNA SChLAP1 Independently Predicts Lethal Prostate Cancer. Eur Urol. 2016;70(4):549-552.

19. Kuner R, Fälth M, Pressinotti NC, et al. The maternal embryonic leucine zipper kinase (MELK) is upregulated in high-grade prostate cancer. J Mol Med (Berl) 2013;91(2):237-48.

20. Penney KL, Sinnott JA, Tyekucheva S, et al. Association of prostate cancer risk variants with gene expression in normal and tumor tissue. Cancer Epidemiol Biomarkers Prev 2015;24(1):255-60.

21. Sinnott JA, Peisch SF, Tyekucheva S, et al. Prognostic Utility of a New mRNA Expression Signature of Gleason Score. Clin Cancer Res 2017;23(1):81-87.

22. Taminau J, Meganck S, Lazar C, et al. Unlocking the potential of publicly available microarray data using inSilicoDb and inSilicoMerging R/Bioconductor packages. BMC Bioinformatics. 2012; 13:335.

23. Feng D, Shi X, Xiong Q, et al. A Gene Prognostic Index Associated With Epithelial-Mesenchymal Transition Predicting Biochemical Recurrence and Tumor Chemoresistance for Prostate Cancer. Front. Oncol. 2022; 11:805571.

24. Bhattacharya S, Dunn P, Thomas CG, et al. ImmPort, toward repurposing of open access immunological assay data for translational and clinical research. Sci Data. 2018; 5:180015.

25. Breuer K, Foroushani AK, Laird MR, et al. InnateDB: systems biology of innate immunity and beyond--recent updates and continuing curation. Nucleic Acids Res. 2013;41(Database issue): D1228-33.

26. Taylor BS, Schultz N, Hieronymus H, et al. Integrative genomic profiling of human prostate cancer. Cancer Cell 2010;18(1):11-22.

27. Friedrich M, Wiedemann K, Reiche K, et al. The Role of lncRNAs TAPIR-1 and -2 as Diagnostic Markers and Potential Therapeutic Targets in Prostate Cancer. Cancers (Basel) 2020;12(5).

28. Liu CJ, Hu FF, Xia MX, et al. GSCALite: a web server for gene set cancer analysis. Bioinformatics. 2018;34(21):3771-3772.

29. Warde-Farley D, Donaldson SL, Comes O, et al. The GeneMANIA prediction server: biological network integration for gene prioritization and predicting gene function. Nucleic Acids Res. 2010;38(Web Server issue):W214-20.

30. Paraskevopoulou MD, Vlachos IS, Karagkouni D, et al. DIANA-LncBase v2: indexing microRNA targets on non-coding transcripts. Nucleic Acids Res. 2016;44(D1): D231-8.

31. Han H, Cho JW, Lee S, et al. TRRUST v2: an expanded reference database of human and mouse transcriptional regulatory interactions. Nucleic Acids Res. 2018;46(D1):D380-D386.

32. Chen Y, Wang X. miRDB: an online database for prediction of functional microRNA targets. Nucleic Acids Res. 2020;48(D1):D127-D131.

33. Liu W, Wang X. Prediction of functional microRNA targets by integrative modeling of microRNA binding and target expression data. Genome Biol. 2019;20(1):18.

34. Subramanian A, Tamayo P, Mootha VK, et al. Gene set enrichment analysis: a knowledge-based approach for interpreting genome-wide expression profiles. Proc Natl Acad Sci U S A. 2005;102(43):15545-50.

35. Liberzon A, Subramanian A, Pinchback R, et al. Molecular signatures database (MSigDB) 3.0. Bioinformatics. 2011;27(12):1739-40.

36. Li Y, Xiao J, Bai J, et al. Molecular characterization and clinical relevance of m6A regulators across 33 cancer types. Mol Cancer. 2019;18(1):137.

37. Zeng D, Ye Z, Shen R, et al. IOBR: Multi-Omics Immuno-Oncology Biological Research to Decode Tumor Microenvironment and Signatures. Front Immunol. 2021; 12:687975.

38. Racle J, de Jonge K, Baumgaertner P, et al. Simultaneous enumeration of cancer and immune cell types from bulk tumor gene expression data. Elife. 2017;6: e26476.

39. Yoshihara K, Shahmoradgoli M, Martínez E, et al. Inferring tumour purity and stromal and immune cell admixture from expression data. Nat Commun. 2013; 4:2612.

40. Charoentong P, Finotello F, Angelova M, et al. Pan-cancer Immunogenomic Analyses Reveal Genotype-Immunophenotype Relationships and Predictors of Response to Checkpoint Blockade. Cell Rep. 2017;18(1):248-262.

41. Shannon P, Markiel A, Ozier O, et al. Cytoscape: a software environment for integrated models of biomolecular interaction networks. Genome Res. 2003;13(11):2498-504.

42. Artibani W, Porcaro AB, De Marco V, et al. Management of Biochemical Recurrence after Primary Curative Treatment for Prostate Cancer: A Review. Urol Int. 2018;100(3):251-262.

43. Markowski MC, Chen Y, Feng Z, et al. PSA doubling time and absolute PSA predict metastasis-free survival in men with biochemically recurrent prostate cancer after radical prostatectomy. Clin Genitourin Cancer. 2019;17(6):470-475.e1.

44. Cornford P, Bellmunt J, Bolla M, et al. EAU-ESTRO-SIOG Guidelines on Prostate Cancer. Part II: Treatment of Relapsing, Metastatic, and Castration-Resistant Prostate Cancer. Eur Urol. 2017;71(4):630-642.

45. Godoy P, Cadenas C, Hellwig B, et al. Interferon-inducible guanylate binding protein (GBP2) is associated with better prognosis in breast cancer and indicates an efficient T cell response. Breast Cancer. 2014;21(4):491-9.

46. Ma J, Sawai H, Matsuo Y, et al. IGF-1 mediates PTEN suppression and enhances cell invasion and proliferation via activation of the IGF-1/PI3K/Akt signaling pathway in pancreatic cancer cells. J Surg Res. 2010;160(1):90-101.

47. Li ZJ, Ying XJ, Chen HL, et al. Insulin-like growth factor-1 induces lymphangiogenesis and facilitates lymphatic metastasis in colorectal cancer. World J Gastroenterol. 2013;19(43):7788-94.

48. Bahhnassy A, Mohanad M, Shaarawy S, et al. Transforming growth factor-β, insulin-like growth factor I/insulin-like growth factor I receptor and vascular endothelial growth factor-A: prognostic and predictive markers in triple-negative and non-triple-negative breast cancer. Mol Med Rep. 2015;12(1):851-64.

49. Erho N, Crisan A, Vergara IA, et al. Discovery and validation of a prostate cancer genomic classifier that predicts early metastasis following radical prostatectomy. PLoS One. 2013;8(6):e66855.

50. Sidiropoulos M, Chang A, Jung K, et al. Expression and regulation of prostate androgen regulated transcript-1 (PART-1) and identification of differential expression in prostatic cancer. Br J Cancer. 2001;85(3):393-7.

51. Sun M, Geng D, Li S, et al. LncRNA PART1 modulates toll-like receptor pathways to influence cell proliferation and apoptosis in prostate cancer cells. Biol Chem. 2018;399(4):387-395.

52. Lou T, Ke K, Zhang L, et al. LncRNA PART1 facilitates the malignant progression of colorectal cancer via miR-150-5p/LRG1 axis. J Cell Biochem. 2020;121(10):4271-4281.

53. Zhou C, Wang P, Tu M, et al. Long Non-Coding RNA PART1 Promotes Proliferation, Migration and Invasion of Hepatocellular Carcinoma Cells via miR-149-5p/MAP2K1 Axis. Cancer Manag Res. 2020; 12:3771-3782.

54. Zhu D, Yu Y, Wang W, et al. Long noncoding RNA PART1 promotes progression of non-small cell lung cancer cells via JAK-STAT signaling pathway. Cancer Med. 2019;8(13):6064-6081.

55. Hanahan D, Weinberg RA. Hallmarks of cancer: the next generation. Cell. 2011;144(5):646-74.

56. Foster DS, Jones RE, Ransom RC, et al. The evolving relationship of wound healing and tumor stroma. JCI Insight. 2018;3(18):e99911.

57. Bonollo F, Thalmann GN, Kruithof-de Julio M, et al. The Role of Cancer-Associated Fibroblasts in Prostate Cancer Tumorigenesis. Cancers (Basel). 2020;12(7):1887.

58. Sahai E, Astsaturov I, Cukierman E, et al. A framework for advancing our understanding of cancer-associated fibroblasts. Nat Rev Cancer. 2020;20(3):174-186.

59. Fiard G, Stavrinides V, Chambers ES, et al. Cellular senescence as a possible link between prostate diseases of the ageing male. Nat Rev Urol. 2021;18(10):597-610.

60. Larionova I, Tuguzbaeva G, Ponomaryova A, et al. Tumor-Associated Macrophages in Human Breast, Colorectal, Lung, Ovarian and Prostate Cancers. Front Oncol. 2020; 10:566511.

61. Zhao SG, Lehrer J, Chang SL, et al. The Immune Landscape of Prostate Cancer and Nomination of PD-L2 as a Potential Therapeutic Target. J Natl Cancer Inst. 2019;111(3):301-310.

**Figure legends**

Figure 1. The flowchart of this study.


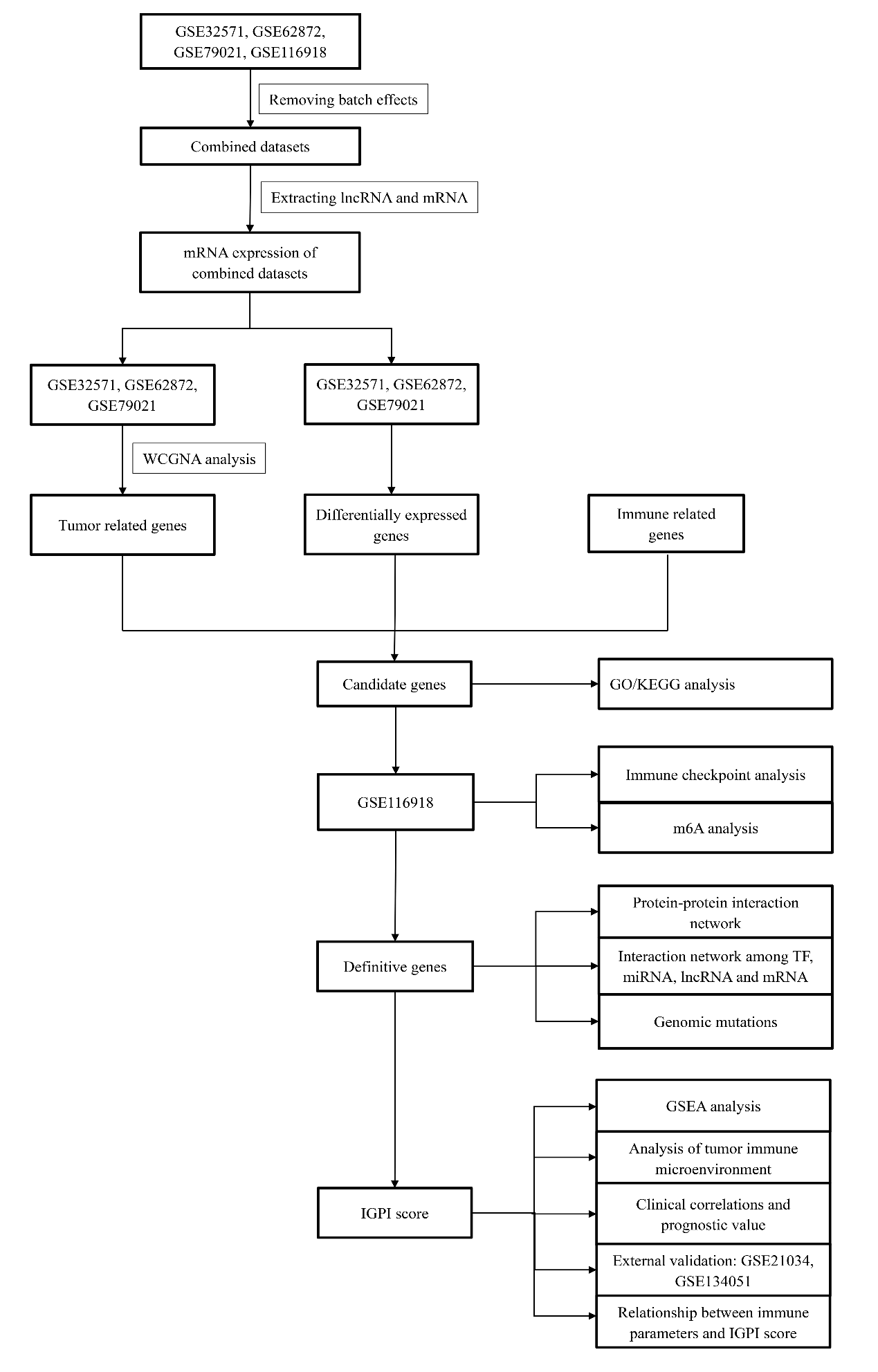


WGCNA= weighted gene co-expression network analysis; GO= gene ontology; KEGG= Kyoto Encyclopedia of Genes and Genome; TF= transcription factor; GSEA= gene set enrichment analysis; IGPI= immunologic gene prognostic index.

Figure 2. The process of definitive genes and interaction networks.


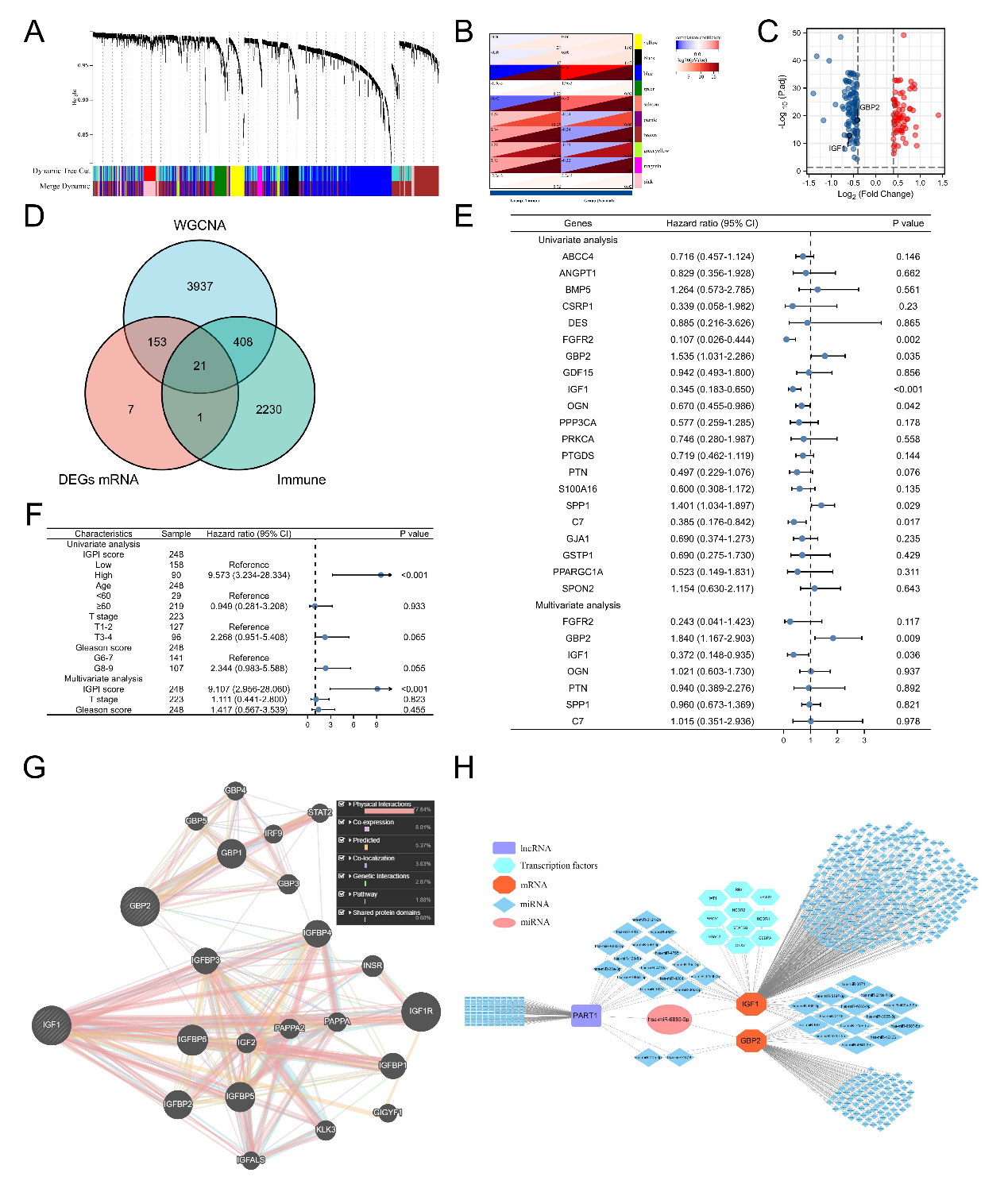


(A) gene cluster; (B) modules and phenotype; (C) volcano plot; (D) Venn diagram; (E) univariate and multivariate COX regression analysis of candidate genes; (F) COX regression analysis of clinical and genomic parameters; (G) protein-protein interaction network; (H) TF and ceRNA network.

Figure 3. Clinical features and values.


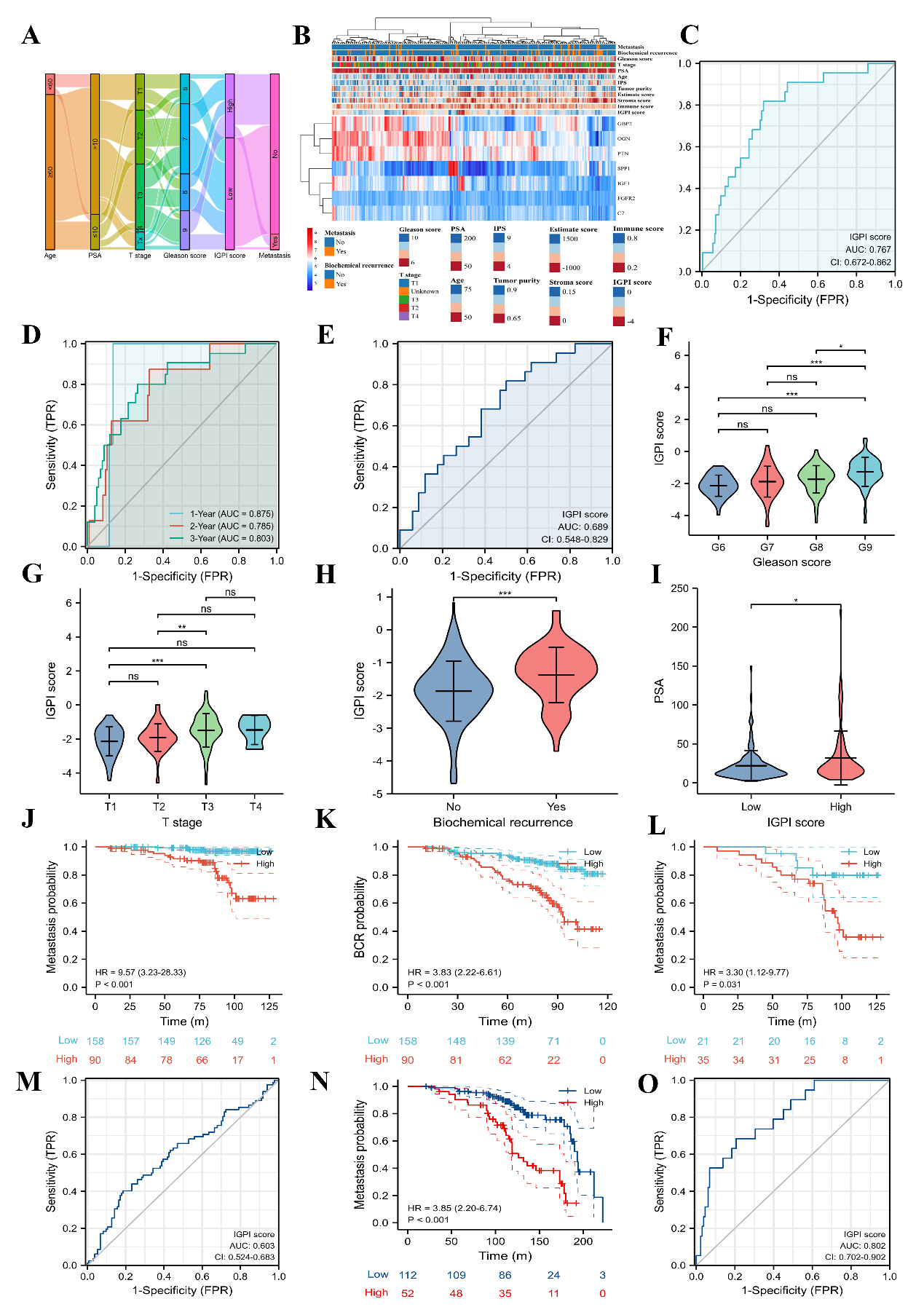


(A) Sankey chart of clinical data, IGPI score and metastasis outcome; (B) heatmap of clinical information, TIME parameters, IGPI score, and metastasis-related DEGs; (C) ROC curve of IGPI score; (D) time-dependent ROC curve of IGPI score; (E) ROC curve of IGPI score after BCR; (F) comparison between Gleason score and IGPI score; (G) comparison between T stage and IGPI score; (H) comparison between biochemical recurrence and IGPI score; (I) comparison between PSA and IGPI score; (J) analysis of metastasis free survival between high and low risk groups; (K) analysis of BCR free survival between high and low risk groups; (L) analysis of metastasis free survival after BCR between high and low risk groups; (M) ROC curve of external validation dataset; (N) analysis of metastasis free survival between high and low risk groups in external validation dataset; (O) ROC curve of another external validation dataset. TIME= tumor immune microenvironment; PSA= prostate specific antigen; DEGs=differentially expressed genes; ROC= receiver operating characteristic; BCR=biochemical recurrence.

Figure 4. Function enrichment analysis.


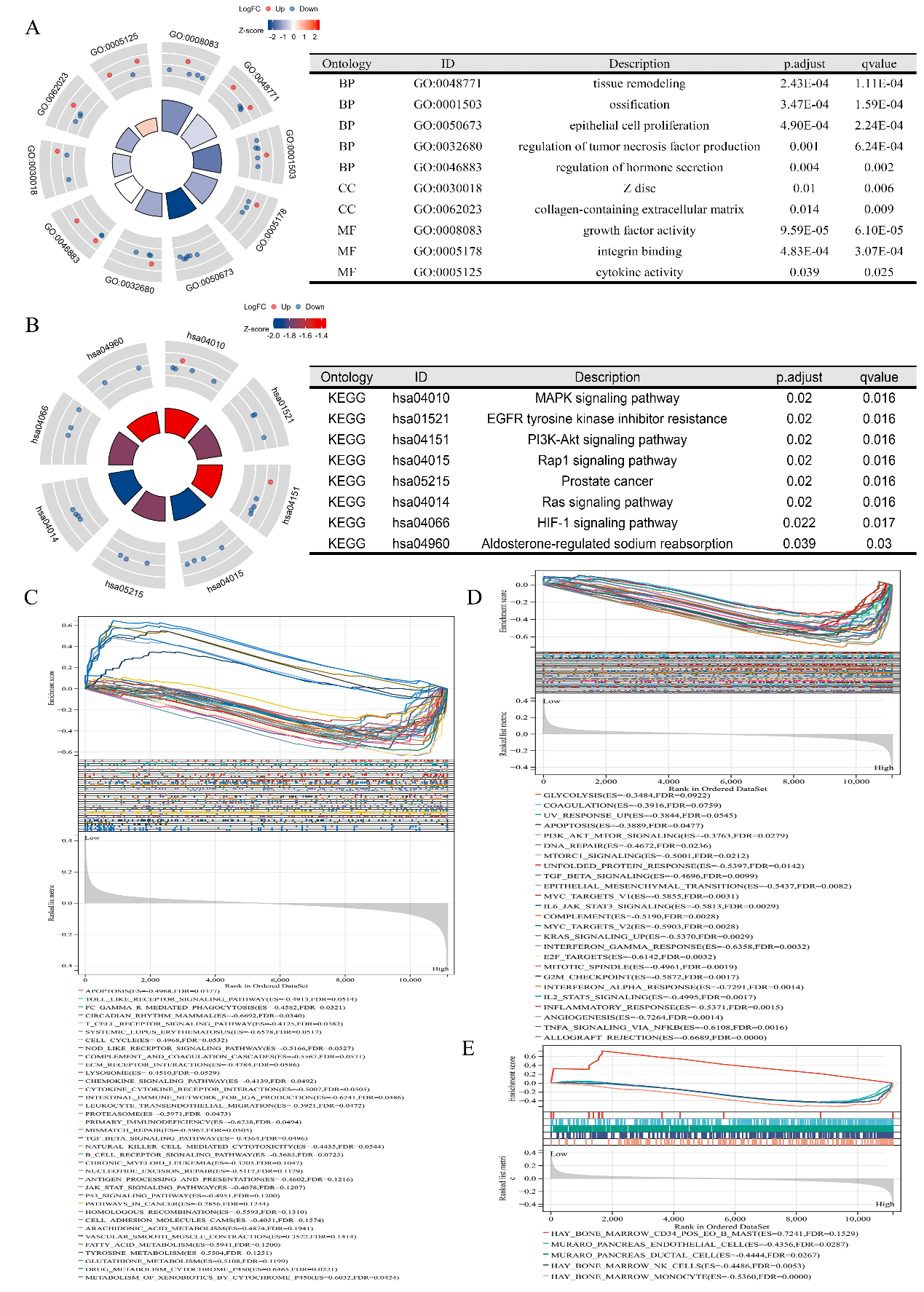


(A) GO analysis; (B) KEGG analysis; (C) GSEA C2 analysis; (D) GSEA C7 analysis; (E) GSEA C8 analysis.

Figure 5. Genomic mutations and tumor immune microenvironment (TIME) analysis.


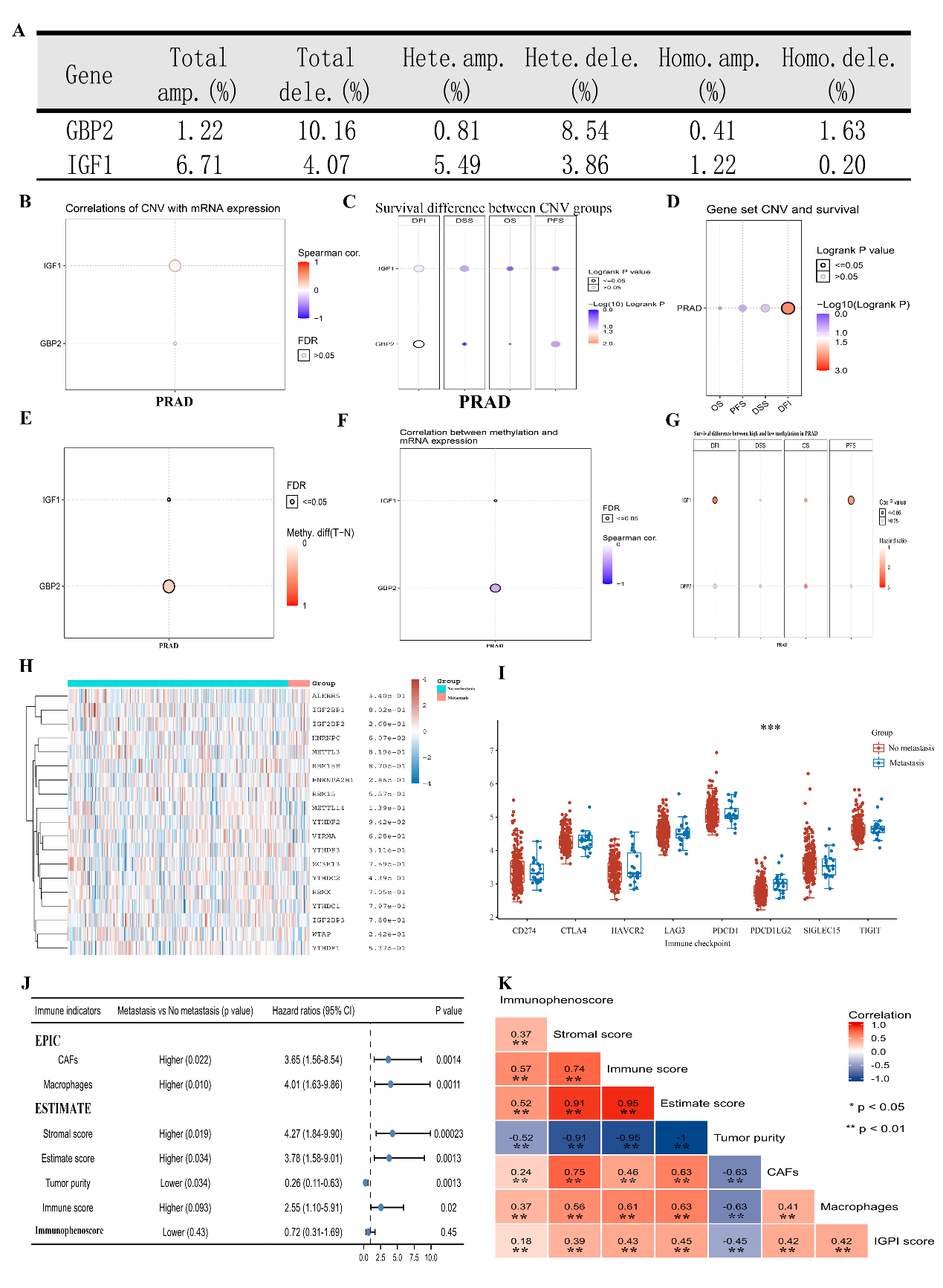


(A) summary of copy number variation (CNV) of GBP2 and IGF1; (B) correlation of CNV with mRNA expression of GBP2 and IGF1 in prostate adenocarcinoma (PRAD); (C) the difference of survival between CNV and wide type in PRAD; (D) the profile of survival between gene set CNV groups in PRAD; (E) the methylation difference between tumor and normal samples of GBP2 and IGF1 in PRAD; (F) correlation between methylation and mRNA expression; (G) survival difference between high and low methylation in PRAD; (H) m6A analysis; (I) immune checkpoint analysis; (J) TIME analysis; (K) heatmap presenting the correlations among immunophenoscore, stromal score, immune score, estimate score, tumor purity, CAFs, macrophages, and IGPI score. Hete. =heterozygous; Homo. =homozygous; Amp. =amplification; Dele. =deletion; DFI=disease free interval; DSS=disease specific survival; OS=overall survival; PFS=progression free survival.
